# P-975. Comprehensive Survey of Current Transplant Infectious Disease Fellowship Programs

**DOI:** 10.1093/ofid/ofae631.1165

**Published:** 2025-01-29

**Authors:** Michael J Scolarici, Jessica S Tischendorf, Rachel Filipiak, Christopher Saddler

**Affiliations:** University of Wisconsin Hospitals and Clinics, Madison, Wisconsin; University of Wisconsin School of Medicine and Public Health, Madision, Wisconsin; University of Wisconsin School of Medicine and Public Health, Madision, Wisconsin; University of Wisconsin, Madison, Wisconsin

## Abstract

**Background:**

Adult Transplant Infectious Disease (TID) training is not ACGME accredited, and there is heterogeneity in training offered at transplant centers.

**Methods:**

The American Society of Transplantation ID Community of Practice surveyed current adult TID fellowship programs and tracks regarding required clinical experiences, non-clinical work, fellowship recruitment/funding, and outcomes of TID fellowship training. Respondents could skip questions, and R was used for statistical analysis.

**Results:**

From 149 adult ID fellowship websites, we identified 37 TID tracks and 25 TID fellowships. After soliciting online forums, we had 24 respondents representing 20 active TID training programs and 16 TID fellowships of which 7/16, 44% have existed for at least 10 years.

In these 16 TID fellowships, fellows averaged 50% of their time with solid organ (SOT) and 41% with hematopoietic stem cell transplantation. The SOT centers were comprehensive (95% offering liver, 95% kidney, 90% heart, 75% lung transplantation). TID fellowships required more inpatient TID consult service compared to TID tracks (28 vs 21 weeks, p=0.1), and TID fellows average 392 (sd=202.8) inpatient TID consults per year. All TID fellowships require TID specific clinic, 93% pre-transplant evaluations, 67% attending selection committees, and 88% scholarship.

All 12 TID fellowships who actively recruit rely on both word of mouth and their program’s website, 11/12, 92% use in-person meetings (e.g. IDWeek), and only 7/12, 58% use social media.

To fund TID fellowships, 11/15, 73% use more than one mechanism (e.g. internal ID division funding, grants), and only 4/15, 27% rely on billable services by TID fellows. Six of 15, 40% hire TID fellows as a clinical instructor/lecturer, the other 9/15, 60% hire the TID fellow as a research/advanced fellow. At 12/14, 86% TID fellowships, more than 50% of the graduates sought TID focused clinical careers. At 6/14, 43% and 11/14, 79% of fellowships none of the graduates sought research focused or industry careers respectively.

**Conclusion:**

TID fellowships require significantly more inpatient consult weeks than tracks, all have non-patient care requirements, the funding for these programs is variable, and most graduates seek a clinically focused career. The heterogeneity of TID fellowships is high.

**Disclosures:**

**Jessica S. Tischendorf, MD, MS**, Merck: Grant/Research Support

